# DNA Methylation: A Key Epigenetic Regulator of Cardiovascular Diseases

**DOI:** 10.31083/RCM46194

**Published:** 2026-04-14

**Authors:** Weihai Chen, Xiangxiang Li, Li Zhu

**Affiliations:** ^1^Department of Cardiology, Suzhou Ninth People's Hospital, Suzhou Ninth Hospital Affiliated to Soochow University, 215200 Suzhou, Jiangsu, China; ^2^Department of Clinical Laboratory, Suzhou Ninth People's Hospital, Suzhou Ninth Hospital Affiliated to Soochow University, 215200 Suzhou, Jiangsu, China; ^3^Laboratories of Thrombosis and Vascular Biology, Suzhou Ninth Hospital Affiliated to Soochow University, 215200 Suzhou, Jiangsu, China

**Keywords:** epigenetics, DNA methylation, cardiovascular risk factors, CVDs, risk prediction, early diagnosis, treatment monitoring

## Abstract

CVDs arise from the interplay of multiple factors, with genetics and environmental exposures representing key drivers. Accumulating evidence over recent decades has unequivocally established that epigenetic modifications, most prominently DNA methylation, play a pivotal, non-redundant role in the initiation, development, and progression of CVDs. As a critical molecular bridge linking genetic predisposition, environmental insults, and the pathogenesis of CVDs, DNA methylation dynamically mediates crosstalk among these three components, thereby emerging as a core focus for dissecting the underlying pathological mechanisms of CVDs. Thus, this review summarizes the functional roles of DNA methylation in common CVDs, including coronary heart disease (CHD), hypertension, and heart failure (HF). Special emphasis is placed on the regulatory mechanisms of DNA methylation in driving disease pathogenesis, as well as the associated translational potential for preventing CVDs and for clinical management. Moreover, this review delineates the specific pathways through which DNA methylation modulates CVDs onset and progression—providing a novel perspective for in-depth investigation of disease etiologies—and offers a robust theoretical basis for identifying novel therapeutic targets for CVDs. Ultimately, these insights aim to lay a foundation for the optimization and innovation of clinical diagnostic and therapeutic strategies for CVDs.

## 1. Introduction 

CVDs have long been the leading cause of death worldwide. The latest statistical data show that the global number of deaths attributed to CVDs reached 20.5 million, accounting for approximately one-third of the total global deaths [1]. This data reflects the severe threat of CVDs to population health on a global scale and underscores the urgency of enhancing disease prevention and control. Although traditional genetic factors and environmental risk factors (such as smoking and unhealthy diet) have been extensively studied, epigenetic mechanisms, particularly DNA methylation, are increasingly emerging as a core link in understanding cardiovascular risk and the development and progression of CVDs. As a “bridge” between environmental and genetic interactions, it profoundly influences disease susceptibility and progression. This article presents a review on the role of DNA methylation in common CVDs such as CHD, atherosclerosis, myocardial infarction, hypertension, HF. It focuses on exploring its regulatory effects in the pathogenesis of these diseases, as well as its application value in the fields of disease prevention and clinical treatment.

## 2. The Basic Concepts and Regulatory Mechanisms of DNA Methylation

Epigenetics refers to a set of specialized molecular mechanisms that regulate gene expression without changing the primary nucleotide sequence of DNA. These mechanisms exert profound effects on cellular function, development processes, and the pathogenesis of diseases. Epigenetics is characterized by two key features: heritability, which allows the transmission of epigenetic marks to daughter cells during cell division or even across generations, and reversibility, which enables dynamic regulatory adaptations in response to environmental stimuli. Together, these properties position epigenetics as a critical bridge connecting genotype and phenotype [2]. The epigenetic regulatory system exhibits high complexity and intricate interconnectivity. Four core classes of epigenetic mechanisms have been extensively investigated: DNA methylation, histone modification, non-coding RNA (ncRNA)-driven regulation, and chromatin remodeling. These regulatory modalities collectively enable spatiotemporally specific gene expression—a process indispensable for cellular differentiation, maintenance of environmental homeostasis, disease progression, and transgenerational phenotypic inheritance [3].

Among diverse epigenetic mechanisms, DNA methylation stands out as the most extensively studied and fundamental one. It dynamically regulates the expression of cardiovascular-associated genes, thereby influencing vascular endothelial function, lipid metabolism, inflammatory responses, and myocardial homeostasis. In turn, this modulation contributes to the initiation, progression, and prognostic outcomes of major CVDs, including CHD, hypertension, HF, and atherosclerosis. Aberrant DNA methylation—encompassing hypermethylation and hypomethylation—of specific genes perturbs the physiological balance of the cardiovascular system, ultimately triggering pathological processes [4]. Notably, such epigenetic abnormalities are amenable to modification by environmental factors (e.g., dietary patterns, tobacco smoking, psychological stress), which further consolidates DNA methylation as a critical mediator linking genetic susceptibility to environmental exposures in the pathogenesis of CVDs.

**Fig. 1.**
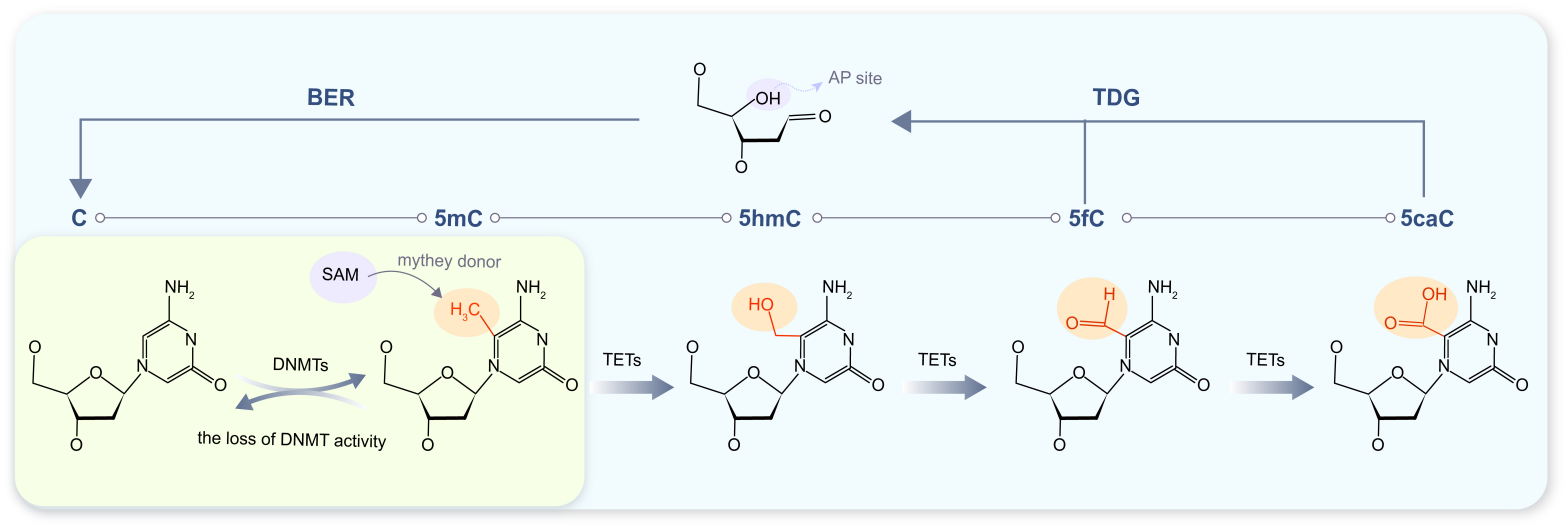
**DNA methylation and demethylation processes**. Regions with a yellow-green background denote DNA methylation and passive demethylation processes, while those with an off-white background correspond to active DNA demethylation. The *TET* protein oxidizes *5mC* to 5-hydroxymethylcytosine (*5hmC*), 5-formylcytosine (*5fC*), and 5-carboxylcytosine (*5caC*) in a sequential, stepwise manner. Notably, *5fC* and *5caC* are unstable intermediates that can be recognized and excised by DNA repair enzymes—including *TDG*. Subsequent coupling with the base excision repair *(BER*) pathway ultimately converts *5mC* back to unmodified cytosine. Abbreviation: *AP* site, apurinic/apyrimidinic site; DNMT(s), DNA methyltransferase(s); SAM, S-adenosylmethionine.

DNA methylation is defined as a chemical modification process wherein a methyl group (CH_3_) is added to the cytosine base of a DNA molecule. This modification predominantly utilizes S-adenosylmethionine (SAM) as the methyl donor, resulting in the formation of 5-methylcytosine (*5mC*) at the 5th carbon position of cytosine residues in double-stranded DNA [5, 6]. The reaction is catalyzed by the DNA methyltransferases (DNMTs) family: specifically, *DNMT3A* and *DNMT3B* mediate “*de novo* methylation” which establishes new methylation patterns [7]; in contrast, *DNMT1* executes “maintenance methylation”, ensuring accurate replication of the original methylation state onto the newly synthesized DNA strand during cell division [8]. Conversely, the erasure of methylation marks depends on the coordinated action of the ten-eleven translocation (*TET*) protein family and thymine DNA glycosylase (*TDG*)—a process termed “demethylation”. Demethylation is categorized into active and passive subtypes. Active demethylation requires the participation of specific enzymes, primarily driven by the *TET* family. Its implementation involves the stepwise oxidation of *5mC*, followed by base excision repair, which ultimately achieves the removal of DNA methyl groups [9, 10]. Passive demethylation, by contrast, is mainly triggered by the loss of DNMT activity; this prevents the newly synthesized DNA strand from retaining the original methylation pattern, thereby leading to the gradual loss of methylation information [11] (Fig. 1).

Notably, this dynamic and reversible modification does not alter the inherent sequence of DNA molecules, but exerts a significant impact on the expression activity of downstream genes by modulating the spatial conformation of chromatin. Generally, a hypermethylated state suppresses gene expression and induces gene silencing, whereas a hypomethylated state tends to enhance gene activation [12]. Thus, sustaining the dynamic equilibrium between methylation and demethylation is an essential prerequisite for preserving genomic stability and ensuring cells correctly specify their fate—an effect with irreplaceable biological significance. In myocardial tissue, DNMTs maintain methylation balance via a multi-node regulatory network, thereby governing the maturation, physiological functions, and pathological responses of cardiomyocytes. In recent years, a growing body of research evidence has demonstrated that aberrant alterations in DNA methylation patterns are closely linked to the pathogenesis of hypertension, atherosclerosis, CHD, and cardiomyopathy, as well as the pathological progression of HF [13, 14].

## 3. The Association Between DNA Methylation and Cardiovascular Disease Risk Factors

The onset of CVDs is typically closely associated with traditional risk factors such as hypertension, dyslipidemia, diabetes mellitus, and obesity. Notably, DNA methylation plays a critical role in the emergence and progression of the aforementioned risk factors, thereby serving as an important “link” that connects environmental exposure to disease onset risk.

### 3.1 Hypertension

Hypertension onset is closely linked to vascular endothelial dysfunction and dysregulation of the renin-angiotensin-aldosterone system (RAAS), among other factors (Table 1).

**Table 1.** **Hypertension-related genes and its epigenetic regulatory role of methylation**.

Hypertension-related genes	Epigenetic regulatory role of methylation
*eNOS*	Hypermethylation of the *eNOS* promoter inhibits *eNOS* transcription and No production, leading to vascular endothelial dysfunction, enhanced vasoconstriction, and increased blood pressure.
*AGT*	Hypomethylation of the *AGT* promoter upregulates *AGT* expression, increasing angiotensin II (Ang II) production, inducing vasoconstriction and sodium-water retention, and elevating blood pressure.
*ATP2B1*	Hypermethylation of the *ATP2B1* promoter inhibits *ATP2B1* expression, reducing renal sodium excretion, enhancing vascular smooth muscle contraction, and elevating blood pressure.

Studies show methylation changes of vascular endothelial function-related genes (e.g., endothelial nitric oxide synthase, *eNOS*) profoundly affect their expression. Specifically, *eNOS* promoter hypermethylation reduces its protein synthesis, causing insufficient nitric oxide (NO) for physiological needs. Nitric oxide synthases *(NOSs*) comprise three isoforms: inducible (*iNOS*), *eNOS*, and neuronal (*nNOS*). *iNOS* is most pathophysiologically relevant—not constitutively expressed but activated/upregulated in states like hypertension or HF [15, 16]. Human/rodent studies confirm *iNOS* transcription is regulated by genomic DNA methylation dynamics, which mediates hypertension pathophysiology [17].

Additionally, the angiotensinogen (*AGT*) gene is core to RAAS; its aberrant methylation alters expression [18, 19]. Angiotensin II (RAAS’s key effector) acts mainly through *AT1R* (with *AT1aR*/*AT1bR* subunits) and *AT2R*. Emerging evidence links *AT1aR* promoter methylation to hypertension [20]. Population studies on *AT1R* methylation and essential hypertension (EH) show EH patients have significantly lower methylation at *AT1R* promoter *CpG1* than healthy controls, suggesting hypomethylation activates *AT1R* transcription, upregulates its mRNA/protein, and promotes hypertension pathogenesis [21].

*ATP2B1* encodes plasma membrane calcium ATPase 1, maintaining intracellular calcium homeostasis, and is validated as a hypertension-susceptible gene by trans-ethnic Genome-Wide Association Study (GWAS). Its epigenetic regulation modulates calcium pump function/vascular physiology, contributing critically to hypertension pathogenesis [22].

### 3.2 Hyperlipidemia

Atherosclerosis development is linked to multiple factors, among which dyslipidemia is widely recognized as a key driver. Within the molecular mechanisms regulating lipid metabolism-related genes, DNA methylation plays a central regulatory role—alterations in its status directly disrupt lipid metabolic balance (Table 2).

**Table 2.** **Hyperlipidemia-related genes and its epigenetic regulatory role of methylation**.

Hyperlipidemia-related genes	Epigenetic regulatory role of methylation
*LDLR*	Hypermethylation of the *LDLR* promoter inhibits gene transcription, reducing hepatic clearance of plasma LDL-C and resulting in hypercholesterolemia.
*ABCG1*	Hypermethylation of the *ABCG1* promoter inhibits *ABCG1* expression, impairs intracellular cholesterol efflux to HDL, induces cholesterol accumulation, and results in hypercholesterolemia.
*PPARG*	Hypermethylation of the *PPARG* promoter inhibits *PPARG* expression, causing dysregulated lipid metabolism and triglyceride accumulation, and resulting in hypercholesterolemia.
*APOE*	Hypermethylation of the *APOE* promoter further exacerbates elevated LDL-C, increasing the risk of hyperlipidemia.

As a core cholesterol metabolism gene, low-density lipoprotein receptor (*LDLR*) encodes a protein that binds circulating low-density lipoprotein cholesterol (LDL-C), facilitating its cellular uptake/degradation to lower plasma LDL-C [23]. *LDLR* promoter hypermethylation represses transcription, reducing protein synthesis [24], impairing LDL-C clearance and causing its vascular intimal accumulation. This accelerates atherosclerotic plaque progression, increases dyslipidemia risk, and elevates subsequent cardiovascular events (e.g., CHD, stroke) [23, 24].

Beyond the *LDLR* gene, aberrant methylation of other lipid transport-associated genes—such as the apolipoprotein B (*ApoB*) gene—can also disrupt normal lipid transport pathways and metabolic processes. This disturbance of systemic lipid homeostasis contributes to the pathological progression of dyslipidemia, thereby participating in atherosclerosis development [25].

*ABCG1* (a key mediator of cholesterol reverse transport) and *PPARG* (a master transcriptional regulator of lipid metabolism) are closely linked to hyperlipidemia via epigenetic regulation. Aberrant promoter hypermethylation of *ABCG1* represses its expression, impairing cholesterol efflux from macrophages and promoting lipid accumulation [26]; hypomethylation of *PPARG* enhances its transcriptional activity, regulating adipogenesis and lipid homeostasis [27]. Dysregulation of these epigenetic modifications disrupts lipid metabolism balance, accelerating the progression of hyperlipidemia and increasing atherosclerotic risk.

### 3.3 Diabetes

Diabetic patients have a much higher risk of CVDs than non-diabetic individuals. DNA methylation is key in type 2 diabetes mellitus (T2DM) pathogenesis and cardiovascular complications [28, 29] (Table 3). Methylation of core insulin signaling gene insulin receptor substrate 1 (*IRS-1*) directly affects insulin signal transduction: its hypermethylation reduces expression, blocking insulin signaling, impairing cellular insulin sensitivity, and inducing insulin resistance—a core pathological link in T2DM [30, 31].

**Table 3.** **Diabetes-related genes and its epigenetic regulatory role of methylation**.

Diabetes-related genes	Epigenetic regulatory role of methylation
*IRS-1*	Hypermethylation of *CpG* sites in the *IRS-1* promoter represses gene transcription, reducing *IRS-1* protein expression in tissues, thereby inhibiting *PI3K*/*Akt* pathway activation, leading to impaired glucose uptake and utilization, and serving as the core pathological basis of type 2 diabetes (T2D).
*PGC-1α*	Hypermethylation of *CpG* sites in the *PGC-1α* promoter represses gene transcription, reducing *PGC-1α* expression in skeletal muscle, liver, pancreas and other tissues, thereby inhibiting insulin signaling transduction, decreasing glucose uptake and utilization in skeletal muscle, promoting hepatic gluconeogenesis, exacerbating insulin resistance, and serving as a core pathological link in type 2 diabetes (T2D).
*PDX-1*	Hypermethylation of *CpG* sites in the *PDX-1* promoter represses gene transcription, markedly reducing *PDX-1* expression in pancreatic β cells, directly impairing their development, proliferation, and survival, repressing insulin gene (*INS*) transcription, and leading to insufficient insulin secretion.
*HNF-4α*	Hypermethylation of *CpG* sites in the *HNF-4α* promoter directly represses gene transcription, reducing *HNF-4α* expression in pancreatic β cells, liver and other tissues, directly repressing insulin gene (*INS*) transcription, impairing the proliferation, survival, and secretory function of pancreatic β cells, leading to insufficient insulin secretion, and serving as a key factor in β-cell failure in type 1 diabetes (T1D) and progression of type 2 diabetes (T2D).

In addition, impaired pancreatic β-cell function with insufficient insulin secretion is another core T2DM pathological driver. Peroxisome proliferator-activated receptor gamma coactivator 1-alpha (PGC-1α), pancreatic and duodenal homeobox 1 (PDX-1), and hepatocyte nuclear factor 4-alpha (HNF-4α) are well-recognized key regulators of β-cell insulin secretion. DNA methylation, a major epigenetic mechanism, profoundly affects their function via expression regulation, contributing to T2DM pathogenesis.

PGC-1α promoter non-CpG methylation is significantly elevated in T2DM skeletal muscle, reducing its mRNA by 40%–60% and mitochondrial DNA copy number by 30% [32]. PDX-1 promoter CpG island methylation (–1200 to –800 bp) is higher in T2DM islets, decreasing its mRNA by 40%–60% and impairing insulin secretion by 30%–50% [33]. Da Li *et al*. [34] reported higher HNF-4α promoter CpG methylation (–1500 to –1200 bp) in T2DM liver, reducing its mRNA by 40%–60%, upregulating gluconeogenic genes (G6PC, PCK1), and elevating fasting blood glucose.

### 3.4 Obesity

The pathogenesis of obesity is closely linked to epigenetic modifications (mainly DNA methylation and histone modifications) of fat mass and obesity-associated protein (*FTO*), melanocortin 4 receptor (*MC4R*), *PPARG*, and leptin/leptin receptor (*LEP*/*LEPR*), whose dysregulation impairs energy metabolism and adipose homeostasis (Table 4). *FTO* promoter hypomethylation—induced by high-fat diet or inactivity—enhances transcription, upregulates *IRX3*/*IRX5*, promotes caloric intake, and inhibits expenditure, causing surplus; hypermethylation silences *FTO* and reduces obesity risk [35, 36]. *MC4R*, a central appetite regulator, is repressed by promoter hypermethylation (blocking transcription factors) or histone deacetylase (*HDAC*)-mediated histone deacetylation, weakening satiety signaling and inducing hyperphagia [37]. *PPARG*, key for adipocyte differentiation, is activated by p300/CBP-mediated acetylation (promoting lipid deposition); aberrant methylation disrupts its regulation of *FABP4*/*AdipoQ*, impairing metabolic balance [38, 39]. *LEP*/*LEPR* epigenetic dysregulation causes leptin resistance: *LEP* hypermethylation inhibits secretion, while *LEPR*’s abnormal H3K4me3 or methylation impairs signaling [40]. These synergistic abnormalities form a “metabolic imbalance-adipose deposition-signaling disorder” cycle, ultimately inducing obesity.

**Table 4.** **Obesity-related genes and its epigenetic regulatory role of methylation**.

Obesity-related genes	Epigenetic regulatory role of methylation
*FTO*	Hypomethylation of the *FTO* promoter markedly upregulates *FTO* expression, which modulates the downstream *IRX3*/*IRX5* gene network, impacts energy expenditure and adipocyte differentiation, and increases individual obesity risk.
*MC4R*	Hypermethylation of the *MC4R* promoter inhibits *MC4R* expression, impairs central appetite-suppressive signaling, and leads to hyperphagia.
*PPARG*	Hypomethylation of the *PPARG* promoter promotes pre-adipocyte differentiation into mature adipocytes, accelerating fat accumulation.
*LEP*/*LEPR*	Hypomethylation of the *LEP* promoter induces leptin overexpression, while hypermethylation of the *LEPR* promoter inhibits its expression, leading to “leptin resistance” and exacerbating energy intake-expenditure imbalance.

## 4. The Role of DNA Methylation in the Onset and Progression of CVDs

Beyond modulating traditional risk factors, DNA methylation also directly contributes to the pathological processes of CVDs—including atherosclerosis, myocardial infarction, and HF—and regulates the initiation and progression of these conditions at the cellular level.

### 4.1 Atherosclerosis

Most CVDs share a common pathological foundation—atherosclerosis—which progresses through a sequence of events: vascular endothelial cell injury, vascular smooth muscle cell (VSMC) proliferation and migration, lipid deposition, and inflammatory response [41]. Upon endothelial cell damage, external stimuli such as oxidative stress and inflammatory factors reduce the methylation levels of adhesion molecule genes (e.g., *VCAM-1*, *ICAM-1*) in endothelial cells, thereby upregulating the expression of these molecules (Table 5). This upregulation promotes leukocyte adhesion to endothelial cells and subsequent migration into the vascular wall, which in turn triggers an inflammatory cascade.

**Table 5.** **Atherosclerosis related genes and its epigenetic regulatory role of methylation**.

Related genes	Epigenetic regulatory role of methylation
*VCAM-1*/*ICAM-1*	Hypomethylation of *CpG* sites in the *VCAM-1*/*ICAM-1* promoter relieves transcriptional repression, markedly upregulating their expression in endothelial and smooth muscle cells, promoting adhesion of monocytes/leukocytes to endothelial cells, accelerating inflammatory cell infiltration, and driving expansion of the plaque lipid core and fibrous cap impairment.
*c-myc*	Hypomethylation of *CpG* sites in the c-myc promoter enhances transcriptional activity, driving cell proliferation, metabolic reprogramming, and inflammatory amplification, and accelerating plaque formation and instability.
*p53*	Hypomethylation of *CpG* sites in the *p53* promoter enhances transcriptional activity, regulating cell apoptosis-proliferation balance, inflammatory and lipid metabolic networks, and exerting a dual regulatory role in plaque formation, progression, and rupture.
*SR-A*	Hypomethylation of *CpG* sites in the *SR-A* promoter enhances transcriptional activity, mediating oxidized low-density lipoprotein (ox-LDL) uptake and activating inflammatory signaling pathways, promoting macrophage foam cell formation and early plaque development, and serving as a key node in crosstalk between lipid metabolism and inflammatory responses.

As the disease advances, VSMCs undergo a phenotypic switch from the contractile to the synthetic phenotype. These phenotypically altered cells proliferate extensively, migrate to the vascular intima, and secrete matrix components (e.g., collagen), leading to fibrous plaque formation [42, 43, 44, 45]. Studies have shown that hypomethylation of proliferation-associated genes (e.g., *c-myc*) in VSMCs activates their expression, thereby driving cell proliferation [45, 46]; conversely, hypermethylation of apoptosis-related genes (e.g., *p53*) suppresses cell apoptosis. This imbalance results in massive VSMC accumulation, further exacerbating plaque formation and progression [47, 48].

Additionally, foam cell formation within plaques is also linked to DNA methylation. Aberrant methylation of the scavenger receptor A (*SR-A*) gene in macrophages modulates its expression, prompting macrophages to phagocytose increased amounts of oxidized low-density lipoprotein (ox-LDL) and differentiate into foam cells—ultimately accelerating lipid core expansion [49, 50].

### 4.2 Myocardial Infarction

Myocardial infarction primarily arises from acute coronary artery occlusion, which induces myocardial cell necrosis via ischemia and hypoxia. Under the stress of myocardial ischemia-hypoxia, the methylation patterns of a subset of genes in myocardial cells undergo rapid alterations—either to adapt to the stressful microenvironment or initiate damage repair mechanisms [51] (Table 6). For example, under normoxic conditions, the promoter region of the hypoxia-inducible factor-1α (*HIF-1α*) gene exhibits a baseline methylation level that restricts its expression; however, upon the onset of myocardial ischemia-hypoxia, *HIF-1α* methylation levels decrease, leading to *HIF-1α* upregulation. This further activates a cascade of downstream genes involved in hypoxia adaptation and angiogenesis (e.g., vascular endothelial growth factor [*VEGF*] gene), promoting collateral circulation formation and attempting to restore myocardial perfusion [52, 53].

**Table 6.** **Myocardial infarction related genes and its epigenetic regulatory role of methylation**.

Related genes	Epigenetic regulatory role of methylation
*HIF-1α*	During myocardial ischemia-hypoxia, the gene’s decreased methylation upregulates *HIF-1α* expression, inducing *VEGF* and promoting aberrant intraplaque neovascularization, increasing vascular permeability and intraplaque hemorrhage risk.
*Bax*	Hypomethylation of *CpG* sites in the *Bax* promoter relieves transcriptional repression, amplifying ischemia-hypoxic myocardial injury via the mitochondrial apoptotic pathway, impairing myocardial repair and ventricular remodeling, with its aberrant activation being a key molecular mechanism for expanded myocardial necrosis and deteriorated cardiac function post-myocardial infarction (MI).
*MMPs*	Enhanced transcription of *MMPs* via their promoters, by degrading the extracellular matrix (ECM), regulating inflammation, and mediating angiogenesis, exerts a “dual role”—moderate activation in the acute phase promotes necrotic tissue clearance and repair, while excessive or sustained activation impairs myocardial structural integrity, exacerbating ventricular dilation and cardiac function deterioration.
*TIMPs*	Hypermethylation of the *TIMPs* promoter represses transcription, specifically inhibiting ECM degradation by *MMPs* (matrix metalloproteinases) and exerting a “protective role” in myocardial repair and structural stability—maintaining ECM integrity and regulating inflammatory repair in the acute phase, while inhibiting excessive remodeling in the chronic phase; insufficient or imbalanced expression exacerbates ventricular dilation and cardiac function deterioration post-myocardial infarction (MI).

Conversely, if ischemia-hypoxia injury is severe, the methylation levels of apoptosis-related genes (e.g., *Bax*) in myocardial cells may decline, resulting in increased *Bax* protein expression, accelerated myocardial cell apoptosis, and infarct size expansion [54, 55]. Additionally, myocardial tissue fibrosis following myocardial infarction is also linked to DNA methylation. Aberrant methylation of genes encoding matrix metalloproteinases (*MMPs*) and their tissue inhibitors (*TIMPs*) disrupts extracellular matrix (ECM) homeostasis, triggers myocardial fibrosis, impairs cardiac function, and elevates the risk of HF [56, 57, 58].

### 4.3 Heart Failure

HF is the end-stage manifestation of various CVDs, with core pathological features of myocardial remodeling and impaired cardiac function. DNA methylation plays a pivotal role in regulating myocardial remodeling, primarily through two key processes: myocardial hypertrophy and myocardial fibrosis (Table 7).

**Table 7.** **HF related genes and its epigenetic regulatory role of methylation**.

Related genes	Epigenetic regulatory role of methylation
*ABP*/*BNP*	Hypomethylation of the *ANP*/*BNP* promoter enhances transcriptional activity, acting as a “compensatory protective factor” released by myocardial stress in HF. Via natriuresis, diuresis, vasodilation, and inhibiting excessive neuroendocrine activation, it delays HF progression and serves as a core biomarker for HF diagnosis and prognosis.
*COL1A1*/*COL3A1*	Decreased methylation of *CpG* sites in the *COL1A1*/*COL3A1* promoter relieves transcriptional repression, activating the genes and inducing excessive collagen synthesis and deposition, leading to myocardial stiffness and ventricular dilation, ultimately exacerbating cardiac function deterioration in HF.
*NET*	Hypermethylation of *CpG* sites in the *NET* promoter represses transcription, hyperactivating myocardial β1-adrenergic receptors, activating downstream *MAPK* and *PI3K*/*Akt* pathways, inducing myocardial hypertrophy and fibrosis, and accelerating ventricular dilation.

Genes linked to myocardial hypertrophy—such as atrial natriuretic peptide (*ANP*) and brain natriuretic peptide (*BNP*)—are weakly expressed in normal myocardial cells. However, during HF progression, hypomethylation of these genes drives their significant upregulation, rendering them key biomarkers for myocardial hypertrophy and cardiac dysfunction. Studies analyzing myocardial tissue samples from HF patients have shown that *ANP* and *BNP* gene methylation levels are significantly lower than those in healthy controls, and their expression levels correlate closely with cardiac function parameters [59, 60].

Meanwhile, aberrant methylation of myocardial fibrosis-associated genes (e.g., type Ⅰ collagen and type Ⅲ collagen genes) triggers excessive collagen synthesis and deposition. This increases myocardial tissue stiffness and impairs ventricular diastolic and systolic function. Relevant studies have demonstrated that, in animal models of myocardial fibrosis, altered methylation levels of type Ⅰ and type Ⅲ collagen genes directly lead to massive collagen accumulation, ultimately causing cardiac dysfunction [61, 62].

Additionally, cardiac sympathetic nerve remodeling is also associated with DNA methylation. Hypermethylation of the norepinephrine transporter (*NET*) gene in sympathetic nerve terminals downregulates *NET* expression, reduces norepinephrine reuptake, and enhances sympathetic nerve excitability—further exacerbating myocardial injury and cardiac function deterioration. A study analyzing sympathetic nerve tissue from HF patients confirmed the association between *NET* gene methylation levels, sympathetic nerve activity, and cardiac function [63].

## 5. The Prospects of DNA Methylation in the Clinical Application of CVDs

In recent years, research exploring the correlation between DNA methylation and CVDs has been continuously deepened. DNA methylation is gradually demonstrating application value in CVD risk assessment, early detection, treatment monitoring, and prognosis evaluation. It is expected to become a novel biomarker and therapeutic target in the field of CVDs, thereby opening up new avenues for achieving precise prevention and treatment of CVDs (Table 8).

**Table 8.** **Methylation markers vs. traditional risk factors: direct comparative evidence for predictive value**.

Methylation markers	Comparison object	Predict the performance of advantages
*ABCG1*	Traditional combination of risk factors	Independently predicts CHD risk with a hazard ratio (HR) of 1.55–1.99, and its predictive power remains unaffected by traditional factors.
*MRS*	Framingham Risk Score	Improved the AUC for acute coronary syndrome (ACS) prediction from 0.72 to 0.83 (+11%) and additionally identified 15% more high-risk individuals.
*LE8*	Traditional risk factors + clinical indicators	Reduced the risk of cardiovascular disease (CVD) by 35% and all-cause mortality by 29%, independently of all traditional risk factors.
*5-CpG*	Traditional risk factors for diabetes	Identified high-risk individuals for future diabetes among obese people with normal blood glucose (risk >20% vs. <5% in the low-risk group).
*ACE*	Blood pressure measurement in the clinic	Predicts hypertension incidence risk and antihypertensive treatment response, offering dynamic monitoring value.

### 5.1 Risk Assessment and Early Diagnosis

Traditionally, risk assessment for CVDs has focused primarily on clinical parameters—including age, gender, blood pressure, blood lipids, and blood glucose. However, the predictive accuracy of this assessment framework has yet to fully meet clinical demands. In contrast, DNA methylation biomarkers offer distinct advantages: they exhibit high specificity, are readily detectable, and can reflect an individual’s genetic susceptibility as well as the long-term cumulative effects of environmental exposures—even prior to disease onset.

For example, measuring the methylation levels of specific genes (e.g., *eNOS*, *LDLR*, *IRS-1*) in blood samples enables assessment of an individual’s risk for hypertension, dyslipidemia, and diabetes, while also predicting the likelihood of subsequent progression to CVDs. This facilitates early disease warning [64]. Notably, in the early stages of atherosclerosis, methylation alterations in relevant genes—either in vascular endothelial cells or circulating blood cells—may precede the emergence of clinical symptoms and imaging findings [65]. One study demonstrated that combining *ANP* and *BNP* methylation patterns improved 1-year mortality prediction in HF patients by 12%, outperforming traditional risk factors alone [66]. A Chinese Han case-control study found *ABCG1*-specific *CpG* methylation was significantly negatively associated with CHD risk, remaining significant after adjusting for confounders (age, gender, hypertension, etc.) and confirming traditional factor-independent value [67]. Another study showed *KCNK3* promoter *rs1275988* methylation correlated with hypertension risk, validated across East Asian and European populations (ethnic generalizability), predicting hypertension earlier than conventional blood pressure measurements (abnormalities detectable in prehypertensives) [68, 69]. Detecting these methylation biomarkers is anticipated to provide a novel strategy for the early diagnosis of CVDs like atherosclerosis, aiding clinicians in intervening earlier in the disease course.

### 5.2 Treatment Monitoring and Prognosis Evaluation

In the management of CVDs, dynamic monitoring of therapeutic efficacy and scientific evaluation of prognosis have long been key priorities for clinicians. Within this process, DNA methylation biomarkers hold considerable value, serving as effective indicators to assess treatment response magnitude and gauge disease prognosis.

For instance, following antihypertensive therapy in hypertensive patients, clinicians can determine treatment effectiveness by measuring changes in *eNOS* gene methylation levels: a reduction in *eNOS* methylation—accompanied by increased *eNOS* expression—often signifies effective treatment and improved vascular endothelial function in the patient. In contrast, if methylation levels fail to change as anticipated (or even persist with insufficient *eNOS* expression), it indicates suboptimal therapeutic efficacy, necessitating re-optimization of the treatment regimen [70, 71]. Additionally, the methylation status of the *ACE* gene (a key component of the RAAS) serves as a supplementary indicator: Clinical studies have shown that patients sensitive to *ACE* inhibitor therapy exhibit moderately increased *ACE* promoter methylation post-treatment, inhibiting excessive gene activation and thereby attenuating vasoconstrictive effects [72]; in contrast, patients with no significant changes in methylation may exhibit drug resistance, requiring a switch in therapeutic targets [73].

In the diagnosis and management of myocardial infarction (MI) patients, detecting methylation levels of genes such as *HIF-1α* and *VEGF* enables clinicians to evaluate myocardial repair potential and collateral circulation establishment—thereby more accurately predicting patients’ prognostic outcomes [74, 75]. Additionally, methylation changes of the apoptosis-related gene *Bax* are a valuable reference: Post-MI *Bax* promoter hypomethylation aggravates cardiomyocyte apoptosis [76]; gradual post-treatment methylation elevation (with inhibited apoptosis) indicates favorable myocardial repair, while persistent hypomethylation predicts expanded necrosis and higher HF risk [77]. Meanwhile, methylation monitoring of fibrosis-related *COL1A1*/*COL3A1* predicts ventricular remodeling—post-treatment methylation elevation reduces collagen synthesis (suppressing remodeling), whereas decreased methylation suggests aggravated fibrosis, requiring intensified antifibrotic therapy [78].

Additionally, in myocardial tissue or blood samples from HF patients, methylation levels of *ANP*, *BNP*, and fibrosis-related genes correlate closely with cardiac function classification and disease progression trends [63]. These markers can be utilized for prognostic risk stratification, providing critical references for adjusting clinical treatment regimens. Additionally, methylation of mitochondrial regulatory gene *PGC-1α* reflects myocardial energy metabolism: *PGC-1α* hypermethylation in HF inhibits mitochondrial biogenesis, worsening energy shortage [79]; post-treatment hypomethylation and gene activation improve contractility and reduce mortality [80]. Meanwhile, methylation monitoring of fibrosis-related genes (e.g., *TGF-β1*, *CTGF*) predicts progression—persistent hypomethylation/upregulation indicates worsening fibrosis, needing timely antifibrotic strategy adjustments [81].

### 5.3 Development of Therapeutic Targets

Given the critical role of DNA methylation in the initiation and progression of CVDs, therapeutic strategies targeting the regulation of DNA methylation processes have emerged as a novel research direction in CVD treatment. Currently, DNA methyltransferase inhibitors (DNMTi) and demethylating agents represent the primary classes of targeted agents that have garnered extensive research interest in this field. These agents exert their effects via two core mechanisms: first, inhibiting the activity of DNA methyltransferases; second, promoting the removal of methyl groups from genes. These actions further alter the methylation status of specific genes, restore their normal expression levels, and ultimately achieve therapeutic outcomes.

For instance, in atherosclerosis therapeutic studies, DNMTi-mediated inhibition of *LDLR* gene hypermethylation can restore the gene’s expression function. This process may enhance the clearance efficiency of circulating LDL-C, thereby retarding atherosclerotic plaque progression [24], but most DNMTi and gene-targeted approaches are in preclinical or very early clinical phases, with important challenges related to specificity, delivery, and long-term safety. In the exploration of treatments for diabetic cardiovascular complications, pharmacologic regulation of *IRS-1* gene methylation levels can improve systemic insulin resistance, which may further mitigate vascular damage induced by hyperglycemia [82].

Additionally, precision methylation regulation technologies targeting specific genes—such as the use of *CRISPR*/*dCas9* to directionally modulate the methylation status of myocardial remodeling-related genes [62]—have theoretically unlocked broader application prospects for precision CVD treatment. Notably, however, most of these targeted therapeutic regimens remain in the stage of basic experimental research or early-phase clinical trials. Their safety and efficacy in clinical settings require further validation through additional subsequent studies.

## 6. Conclusions and Outlook

As a key epigenetic regulatory mechanism, DNA methylation exerts a central role in the development of CVD risk factors and the entire trajectory of disease initiation and progression by precisely regulating the expression of cardiovascular-associated genes. Evidence of DNA methylation involvement is observable not only in the regulatory cascades of foundational risk factors (e.g., hypertension, dyslipidemia, diabetes) but also in the pathological progression of conditions such as atherosclerosis, myocardial infarction, and HF. Concurrently, DNA methylation holds substantial clinical application potential in CVD risk prediction, early detection, therapeutic efficacy monitoring, and prognostic assessment—providing novel insights and research avenues for the precision prevention and management of these diseases.

Large-scale tissue-specific CVD methylation profiles are the cornerstone for epigenetic mechanism elucidation and precision biomarker screening, with preliminary data: vascular tissues focus on differential methylation of atherosclerosis-related genes (e.g., *eNO*S, *ABCG1*) [83]; myocardial tissues clarify methylation associations of *ANP*/*BNP* family/fibrosis-related genes with cardiac function [84]; peripheral blood leukocytes serve as convenient surrogates for large-scale studies [85, 86]. Functional *CpG*-derived multi-marker methylation panels, validated in prospective cohorts, outperform traditional risk factors and single biomarkers—CHD panel (8 *CpG* sites, including *ABCG1*/*eNOS*) achieved AUC = 0.83 (10-year incident risk, European cohorts, +15% vs. Framingham) [87]; HF prognosis panel (12 *CpG* sites) showed HR = 2.89 (95% CI: 2.13–3.92, 5-year post-MI HF, North American cohorts) with higher accuracy than NT-proBNP [88]. Furthermore, personalized epigenetic therapy/epigenome editing enables “tailored” intervention via precise regulation of aberrant methylation sites (e.g., *ABCG1* hypomethylation in CHD, *COL1A1* hypermethylation in fibrosis), forming a complete translational chain from mechanism to prediction and precision intervention [89].

Nevertheless, current research on DNA methylation and CVDs still confronts multiple challenges. First, DNA methylation patterns exhibit marked variability across different tissues and cell types, and further investigation is required to identify how to select appropriate detection samples (e.g., blood, tissue, or bodily fluid specimens) to accurately reflect the actual disease state. Second, CVD occurrence arises from the synergistic action of multiple genes and factors; thus, relying solely on a single methylation biomarker for diagnosis and prediction offers relatively limited utility. Consequently, screening and validating more methylation biomarker panels with both specificity and sensitivity has become an urgent unmet need. Additionally, clinically applied therapeutic agents targeting DNA methylation may induce off-target effects and potential toxicities. Enhancing the targeting precision and safety of these agents represents another key challenge to be addressed in future research.

With the continuous innovation of epigenetic research technologies and the widespread adoption of techniques such as high-throughput sequencing and single-cell methylation sequencing, we will gain the opportunity to dissect the mechanism of DNA methylation in CVDs more comprehensively and deeply. This will facilitate the discovery of additional clinically valuable methylation biomarkers and the development of safer, more effective targeted therapeutics—ultimately advancing the prevention and treatment of CVDs into a new developmental phase.

## References

[1] World Heart Federation (2023). World Heart Report 2023: Confronting the World’s Number One Killer.

[2] John RM, Rougeulle C (2018). Developmental Epigenetics: Phenotype and the Flexible Epigenome. *Frontiers in Cell and Developmental Biology*.

[3] Atabekov T, Korepanov V, Krivolapov S, Khlynin M, Afanasiev S (2025). Mitochondrial DNA Polymorphisms of Peripheral Blood Mononuclear Cells Associated with Sustained Ventricular Tachycardia in Patients with Cardioverter-Defibrillator Implantation Indications. *Reviews in Cardiovascular Medicine*.

[4] Burgess DJ (2023). Engineering transgenerational epigenetic inheritance in mammals. *Nature Reviews. Genetics*.

[5] Greenberg MVC, Bourc’his D (2019). The diverse roles of DNA methylation in mammalian development and disease. *Nature Reviews. Molecular Cell Biology*.

[6] Schulz M, Teissandier A, De La Mata Santaella E, Armand M, Iranzo J, El Marjou F (2024). DNA methylation restricts coordinated germline and neural fates in embryonic stem cell differentiation. *Nature Structural & Molecular Biology*.

[7] Li Y, Chen X, Lu C (2021). The interplay between DNA and histone methylation: molecular mechanisms and disease implications. *EMBO Reports*.

[8] Chattopadhyaya S, Ghosal S (2022). DNA methylation: a saga of genome maintenance in hematological perspective. *Human Cell*.

[9] Zhang X, Zhang Y, Wang C, Wang X (2023). TET (Ten-eleven translocation) family proteins: structure, biological functions and applications. *Signal Transduction and Targeted Therapy*.

[10] Wu X, Zhang Y (2017). TET-mediated active DNA demethylation: mechanism, function and beyond. *Nature Reviews. Genetics*.

[11] Moore LD, Le T, Fan G (2013). DNA methylation and its basic function. *Neuropsychopharmacology: Official Publication of the American College of Neuropsychopharmacology*.

[12] Portela A, Esteller M (2010). Epigenetic modifications and human disease. *Nature Biotechnology*.

[13] Tan K, Foo R, Loh M (2023). Cardiomyopathy in Asian Cohorts: Genetic and Epigenetic Insights. *Circulation. Genomic and Precision Medicine*.

[14] Sabia C, Picascia A, Grimaldi V, Amarelli C, Maiello C, Napoli C (2017). The epigenetic promise to improve prognosis of HF and heart transplantation. *Transplantation Reviews (Orlando, Fla.)*.

[15] Hong HJ, Loh SH, Yen MH (2000). Suppression of the development of hypertension by the inhibitor of inducible nitric oxide synthase. *British Journal of Pharmacology*.

[16] Feng Q, Lu X, Jones DL, Shen J, Arnold JM (2001). Increased inducible nitric oxide synthase expression contributes to myocardial dysfunction and higher mortality after myocardial infarction in mice. *Circulation*.

[17] Chan GC, Fish JE, Mawji IA, Leung DD, Rachlis AC, Marsden PA (2005). Epigenetic basis for the transcriptional hyporesponsiveness of the human inducible nitric oxide synthase gene in vascular endothelial cells. *Journal of Immunology (Baltimore, Md.: 1950)*.

[18] Savoia C, Burger D, Nishigaki N, Montezano A, Touyz RM (2011). Angiotensin II and the vascular phenotype in hypertension. *Expert Reviews in Molecular Medicine*.

[19] Navar LG, Prieto MC, Satou R, Kobori H (2011). Intrarenal angiotensin II and its contribution to the genesis of chronic hypertension. *Current Opinion in Pharmacology*.

[20] Pei F, Wang X, Yue R, Chen C, Huang J, Huang J (2015). Differential expression and DNA methylation of angiotensin type 1A receptors in vascular tissues during genetic hypertension development. *Molecular and Cellular Biochemistry*.

[21] Fan R, Mao S, Zhong F, Gong M, Yin F, Hao L (2015). Association of AGTR1 Promoter Methylation Levels with Essential Hypertension Risk: A Matched Case-Control Study. *Cytogenetic and Genome Research*.

[22] Wang Y, Zhang Y, Li Y, Zhou X, Wang X, Gao P (2013). Common variants in the ATP2B1 gene are associated with hypertension and arterial stiffness in Chinese population. *Molecular Biology Reports*.

[23] Brown MS, Goldstein JL (1986). A receptor-mediated pathway for cholesterol homeostasis. *Science (New York, N.Y.)*.

[24] Zorzo RA, Suen VMM, Santos JE, Silva-Jr WA, Suazo VK, Honorato ALSC (2023). LDLR gene’s promoter region hypermethylation in patients with familial hypercholesterolemia. *Scientific Reports*.

[25] Westerman KE, Ordovás JM (2020). DNA methylation and incident cardiovascular disease. *Current Opinion in Clinical Nutrition and Metabolic Care*.

[26] Zhou W, Sun J, Huai C, Liu Y, Chen L, Yi Z (2022). Multi-omics analysis identifies rare variation in leptin/PPAR gene sets and hypermethylation of ABCG1 contribute to antipsychotics-induced metabolic syndromes. *Molecular Psychiatry*.

[27] Liu Y, He T, Li Z, Sun Z, Wang S, Shen H (2023). TET2 is recruited by CREB to promote Cebpb, Cebpa, and Pparg transcription by facilitating hydroxymethylation during adipocyte differentiation. *iScience*.

[28] Hillary RF, McCartney DL, Smith HM, Bernabeu E, Gadd DA, Chybowska AD (2023). Blood-based epigenome-wide analyses of 19 common disease states: A longitudinal, population-based linked cohort study of 18,413 Scottish individuals. *PLoS Medicine*.

[29] Liu Z, Zhang Y, Qiu C, Zhu H, Pan S, Jia H (2020). Diabetes mellitus exacerbates post-myocardial infarction HF by reducing sarcolipin promoter methylation. *ESC HF*.

[30] Liu HW, Mahmood S, Srinivasan M, Smiraglia DJ, Patel MS (2013). Developmental programming in skeletal muscle in response to overnourishment in the immediate postnatal life in rats. *The Journal of Nutritional Biochemistry*.

[31] Tian M, Han YB, Zhao CC, Liu L, Zhang FL (2021). Hesperidin alleviates insulin resistance by improving HG-induced oxidative stress and mitochondrial dysfunction by restoring miR-149. *Diabetology & Metabolic Syndrome*.

[32] Barrès R, Osler ME, Yan J, Rune A, Fritz T, Caidahl K (2009). Non-CpG methylation of the PGC-1alpha promoter through DNMT3B controls mitochondrial density. *Cell Metabolism*.

[33] Yang BT, Dayeh TA, Volkov PA, Kirkpatrick CL, Malmgren S, Jing X (2012). Increased DNA methylation and decreased expression of PDX-1 in pancreatic islets from patients with type 2 diabetes. *Molecular Endocrinology (Baltimore, Md.)*.

[34] Da Li, Cao T, Sun X, Jin S, Di Xie, Huang X (2020). Hepatic TET3 contributes to type-2 diabetes by inducing the HNF4α fetal isoform. *Nature Communications*.

[35] Church C, Moir L, McMurray F, Girard C, Banks GT, Teboul L (2010). Overexpression of Fto leads to increased food intake and results in obesity. *Nature Genetics*.

[36] Sobreira DR, Joslin AC, Zhang Q, Williamson I, Hansen GT, Farris KM (2021). Extensive pleiotropism and allelic heterogeneity mediate metabolic effects of IRX3 and IRX5. *Science*.

[37] Tang Y, Jin B, Zhou L, Lu W (2017). MeQTL analysis of childhood obesity links epigenetics with a risk SNP rs17782313 near MC4R from meta-analysis. *Oncotarget*.

[38] Li Q, Peng H, Fan H, Zou X, Liu Q, Zhang Y (2016). The LIM protein Ajuba promotes adipogenesis by enhancing PPARγ and p300/CBP interaction. *Cell Death and Differentiation*.

[39] Małodobra-Mazur M, Cierzniak A, Kaliszewski K, Dobosz T (2021). PPARG Hypermethylation as the First Epigenetic Modification in Newly Onset Insulin Resistance in Human Adipocytes. *Genes*.

[40] Kalashikam RR, Inagadapa PJN, Thomas AE, Jeyapal S, Giridharan NV, Raghunath M (2014). Leptin gene promoter DNA methylation in WNIN obese mutant rats. *Lipids in Health and Disease*.

[41] Mach F, Baigent C, Catapano AL, Koskinas KC, Casula M, Badimon L (2020). 2019 ESC/EAS Guidelines for the management of dyslipidaemias: lipid modification to reduce cardiovascular risk. *European Heart Journal*.

[42] Dunn J, Qiu H, Kim S, Jjingo D, Hoffman R, Kim CW (2014). Flow-dependent epigenetic DNA methylation regulates endothelial gene expression and atherosclerosis. *The Journal of Clinical Investigation*.

[43] Hu C, Peng K, Wu Q, Wang Y, Fan X, Zhang DM (2021). HDAC1 and 2 regulate endothelial VCAM-1 expression and atherogenesis by suppressing methylation of the GATA6 promoter. *Theranostics*.

[44] Marzolla V, Armani A, Mammi C, Moss ME, Pagliarini V, Pontecorvo L (2017). Essential role of ICAM-1 in aldosterone-induced atherosclerosis. *International Journal of Cardiology*.

[45] Zhang Y, Mei J, Li J, Zhang Y, Zhou Q, Xu F (2021). DNA Methylation in Atherosclerosis: A New Perspective. *Evidence-based Complementary and Alternative Medicine: ECAM*.

[46] Huang S, Shao T, Liu H, Wang Q, Li T, Zhao Q (2022). SIRT6 mediates MRTF-A deacetylation in vascular endothelial cells to antagonize oxLDL-induced ICAM-1 transcription. *Cell Death Discovery*.

[47] Zhu L, Jia L, Liu N, Wu R, Guan G, Hui R (2022). DNA Methyltransferase 3b Accelerates the Process of Atherosclerosis. *Oxidative Medicine and Cellular Longevity*.

[48] Wei Y, Sun Z, Wang Y, Xie Z, Xu S, Xu Y (2019). Methylation in the TP53 promoter is associated with ischemic stroke. *Molecular Medicine Reports*.

[49] Li Y, Zhou M, Li H, Dai C, Yin L, Liu C (2024). Macrophage P2Y6 receptor deletion attenuates atherosclerosis by limiting foam cell formation through phospholipase Cβ/store-operated calcium entry/calreticulin/scavenger receptor A pathways. *European Heart Journal*.

[50] Liu X, Guo JW, Lin XC, Tuo YH, Peng WL, He SY (2021). Macrophage NFATc3 prevents foam cell formation and atherosclerosis: evidence and mechanisms. *European Heart Journal*.

[51] Li M, Jiao L, Shao Y, Li H, Sun L, Yu Q (2022). LncRNA-ZFAS1 Promotes Myocardial Ischemia-Reperfusion Injury Through DNA Methylation-Mediated Notch1 Down-Regulation in Mice. *JACC. Basic to Translational Science*.

[52] Luo X, Hu Y, Shen J, Liu X, Wang T, Li L (2022). Integrative analysis of DNA methylation and gene expression reveals key molecular signatures in acute myocardial infarction. *Clinical Epigenetics*.

[53] Zhang YZ, Lin TT, Fan SM, Wu YQ (2025). Dapagliflozin Suppressed Cuproptosis and Myocardial Fibrosis in Myocardial Infarction Through HIF-1α/TGF-β Pathway. *Current Medical Science*.

[54] Przepiórska K, Wnuk A, Beyer C, Kajta M (2023). Amorfrutin B Protects Mouse Brain Neurons from Hypoxia/Ischemia by Inhibiting Apoptosis and Autophagy Processes Through Gene Methylation- and miRNA-Dependent Regulation. *Molecular Neurobiology*.

[55] Hochhauser E, Cheporko Y, Yasovich N, Pinchas L, Offen D, Barhum Y (2007). Bax deficiency reduces infarct size and improves long-term function after myocardial infarction. *Cell Biochemistry and Biophysics*.

[56] Chaturvedi P, Tyagi SC (2016). Epigenetic silencing of TIMP4 in HF. *Journal of Cellular and Molecular Medicine*.

[57] Zhang X, Hu M, Lyu X, Li C, Thannickal VJ, Sanders YY (2017). DNA methylation regulated gene expression in organ fibrosis. *Biochimica et Biophysica Acta. Molecular Basis of Disease*.

[58] Kou S, Lu Z, Deng D, Ye M, Sui Y, Qin L (2025). Activation of Imprinted Gene PW1 Promotes Cardiac Fibrosis After Ischemic Injury. *Circulation*.

[59] Inazumi H, Kuwahara K (2022). NRSF/REST-Mediated Epigenomic Regulation in the Heart: Transcriptional Control of Natriuretic Peptides and Beyond. *Biology*.

[60] Oeing CU, Pepin ME, Saul KB, Agircan AS, Assenov Y, Merkel TS (2023). Indirect epigenetic testing identifies a diagnostic signature of cardiomyocyte DNA methylation in HF. *Basic Research in Cardiology*.

[61] Pan X, Chen Z, Huang R, Yao Y, Ma G (2013). Transforming growth factor β1 induces the expression of collagen type I by DNA methylation in cardiac fibroblasts. *PloS One*.

[62] Li X, Yang Y, Chen S, Zhou J, Li J, Cheng Y (2021). Epigenetics-based therapeutics for myocardial fibrosis. *Life Sciences*.

[63] Marín-García J, Akhmedov AT (2015). Epigenetics of the failing heart. *HF Reviews*.

[64] Palou-Márquez G, Subirana I, Nonell L, Fernández-Sanlés A, Elosua R (2021). DNA methylation and gene expression integration in cardiovascular disease. *Clinical Epigenetics*.

[65] Jin J, Zhao X, Zhu C, Li M, Wang J, Fan Y (2023). Hypomethylation of ABCG1 in peripheral blood as a potential marker for the detection of CHD. *Clinical Epigenetics*.

[66] Rubattu S, Stanzione R, Cotugno M, Bianchi F, Marchitti S, Forte M (2020). Epigenetic control of natriuretic peptides: implications for health and disease. *Cellular and Molecular Life Sciences: CMLS*.

[67] Qie R, Chen Q, Wang T, Chen X, Wang J, Cheng R (2021). Association of ABCG1 gene methylation and its dynamic change status with incident type 2 diabetes mellitus: the Rural Chinese Cohort Study. *Journal of Human Genetics*.

[68] Huang D, Shang W, Xu M, Wan Q, Zhang J, Tang X (2024). Genome-Wide Methylation Analysis Reveals a KCNK3-Prominent Causal Cascade on Hypertension. *Circulation Research*.

[69] Avila Martins CC, Maschietto M, Kimura L, Alvizi L, Nunes K, Magalhães Borges V (2025). Differential methylation in blood pressure control genes is associated to essential hypertension in African Brazilian populations. *Epigenetics*.

[70] Tang B, Li X, Wang Y, Sjölander A, Johnell K, Thambisetty M (2023). Longitudinal associations between use of antihypertensive, antidiabetic, and lipid-lowering medications and biological aging. *GeroScience*.

[71] Amenyah SD, Ward M, McMahon A, Deane J, McNulty H, Hughes C (2021). DNA methylation of hypertension-related genes and effect of riboflavin supplementation in adults stratified by genotype for the MTHFR C677T polymorphism. *International Journal of Cardiology*.

[72] Bai C, Su M, Zhang Y, Lin Y, Sun Y, Song L (2022). Oviductal Glycoprotein 1 Promotes Hypertension by Inducing Vascular Remodeling Through an Interaction With MYH9. *Circulation*.

[73] Danilov SM, Tovsky SI, Schwartz DE, Dull RO (2017). ACE Phenotyping as a Guide Toward Personalized Therapy With ACE Inhibitors. *Journal of Cardiovascular Pharmacology and Therapeutics*.

[74] Turunen MP, Husso T, Musthafa H, Laidinen S, Dragneva G, Laham-Karam N (2014). Epigenetic upregulation of endogenous VEGF-A reduces myocardial infarct size in mice. *PloS One*.

[75] Zemmour H, Planer D, Magenheim J, Moss J, Neiman D, Gilon D (2018). Non-invasive detection of human cardiomyocyte death using methylation patterns of circulating DNA. *Nature Communications*.

[76] Bi F, Cao M, Wang Y, Pan Q, Jing Z, Bing D (2024). YBX1 inhibits mitochondrial-mediated apoptosis in ischemic heart through the PI3K/AKT signaling pathway. *Frigid Zone Medicine*.

[77] Liu L, Yu L, Wang Y, Zhou L, Liu Y, Pan X (2024). Unravelling the impact of RNA methylation genetic and epigenetic machinery in the treatment of cardiomyopathy. *Pharmacological Research*.

[78] Węgiel M, Surmiak M, Malinowski KP, Dziewierz A, Surdacki A, Bartuś S (2024). In-Hospital Levels of Circulating MicroRNAs as Potential Predictors of Left Ventricular Remodeling Post-Myocardial Infarction. *Medicina (Kaunas, Lithuania)*.

[79] Oka SI, Sabry AD, Cawley KM, Warren JS (2020). Multiple Levels of PGC-1α Dysregulation in HF. *Frontiers in Cardiovascular Medicine*.

[80] Desiderio A, Pastorino M, Campitelli M, Longo M, Miele C, Napoli R (2024). DNA methylation in cardiovascular disease and HF: novel prediction models?. *Clinical Epigenetics*.

[81] Zhang Y, Yan H, Guang GC, Deng ZR (2017). Overexpressed connective tissue growth factor in cardiomyocytes attenuates left ventricular remodeling induced by angiotensin II perfusion. *Clinical and Experimental Hypertension (New York, N.Y.: 1993)*.

[82] You D, Nilsson E, Tenen DE, Lyubetskaya A, Lo JC, Jiang R (2017). Dnmt3a is an epigenetic mediator of adipose insulin resistance. *eLife*.

[83] Carbonneau M, Li Y, Qu Y, Zheng Y, Wood AC, Wang M (2025). DNA Methylation Signatures of Cardiovascular Health Provide Insights Into Diseases. *Circulation*.

[84] Song W, Wang H, Wu Q (2015). Atrial natriuretic peptide in cardiovascular biology and disease (NPPA). *Gene*.

[85] Cuadrat RRC, Kratzer A, Arnal HG, Rathgeber AC, Wreczycka K, Blume A (2023). Cardiovascular disease biomarkers derived from circulating cell-free DNA methylation. *NAR Genomics and Bioinformatics*.

[86] Zuo HP, Guo YY, Che L, Wu XZ (2016). Hypomethylation of Interleukin-6 Promoter is Associated with the Risk of CHD. *Arquivos Brasileiros De Cardiologia*.

[87] Huan T, Joehanes R, Song C, Peng F, Guo Y, Mendelson M (2019). Genome-wide identification of DNA methylation QTLs in whole blood highlights pathways for cardiovascular disease. *Nature Communications*.

[88] Lin Z, Chang J, Li X, Wang J, Wu X, Liu X (2022). Association of DNA methylation and transcriptome reveals epigenetic etiology of HF. *Functional & Integrative Genomics*.

[89] Holmes MV, Richardson TG, Ference BA, Davies NM, Davey Smith G (2021). Integrating genomics with biomarkers and therapeutic targets to invigorate cardiovascular drug development. *Nature Reviews. Cardiology*.

